# Metformin inhibits urothelial tumorigenesis in the UPII-mutant Ha-ras transgenic mouse model

**DOI:** 10.1186/2049-3002-2-S1-P90

**Published:** 2014-05-28

**Authors:** Zhongbo Liu, Noriko Yokoyama, Michael Pollak, Xiaolin Zi

**Affiliations:** 1 Urology, University of California,Irvine, Orange, CA, USA; 2 Oncology, McGill University, Montreal, Quebec, Canada; 3 Urology, Pharmacology and Pharmaceutical Sciences, University of California, Irvine, Orange, CA, USA

## Background

Bladder cancer occurs mainly in older people and is expensive to manage. As the population continues to age, bladder cancer may remain to be a major public health burden. Therefore, we hypothesized that an agent, like metformin, with anti-aging property could have preventive activity against the development and progression of human urinary bladder cancer.

## Materials and methods

The UPII mutant Ha-ras transgenic mouse model mimics human papillary transitional urothelial cell carcinoma and exhibits enhanced mTOR activity in tumor tissues. Homozygous UPII mutant Ha-ras transgenic mice were identified through genotyping using the Southern blotting method. Genotyped Ha-ras mice were then fed orally with normal drinking water or 0.1% or 0.05% metformin in drinking water starting at 6 weeks of age and ending at 6 months of age. Death rate, body weight, tumor burden, and proliferative and apoptotic indices at the end of treatments will be evaluated by pathological and statistical analyses.

## Results

About 62% of male, homozygous mutant Ha-ras transgenic mice which drank normal water died of urinary tract obstruction and hydronephrosis within 6 months of age, while only about 11% or 15% of mice which drank 0.1% or 0.05% metformin containing water died. Drinking metformin dramatically increased the survival of mutant Ha-ras tumor bearing mice by 51 to 47% (Ps<0.01). Metformin drinking also significantly decreased the mean bladder weights of male, homozygous mutant Ha-ras transgenic mice by up to 62%. Histological analysis of H&E stained bladder sections from metformin treated mice demonstrated more differentiated tumors compared to those in control groups. The *in vivo* mechanisms of metformin’s action are associated with anti-proliferation, reduction of phospho-mTOR and 4E-BP1 expression and induction of TSC2 expression in bladder tissues.

## Conclusions

Our results demonstrated strong *in vivo* anti-urothelial tumorigenesis activity of metformin drinking in the UPII-mutant H-ras model via inhibition of the mTOR pathway. These results suggested the potential of metformin in preventing recurrence of clinical bladder cancer and in improving quality of life for patients.

